# Ginsenoside Rg1 as a promising adjuvant agent for enhancing the anti-cancer functions of granulocytes inhibited by noradrenaline

**DOI:** 10.3389/fimmu.2023.1070679

**Published:** 2023-02-01

**Authors:** Yuqian Zhu, Jingyao Chen, Jun Li, Chenqi Zhou, Xin Huang, Bingdi Chen

**Affiliations:** Shanghai East Hospital, The Institute for Biomedical Engineering & Nano Science, Tongji University School of Medicine, Shanghai, China

**Keywords:** granulocytes, cancer, ginsenoside Rg1, noradrenaline, stress

## Abstract

**Introduction:**

In recent years, numerous studies have confirmed that chronic stress is closely related to the development of cancer. Our previous research showed that high levels of stress hormones secreted in the body during chronic stress could inhibit the cancer-killing activity of granulocytes, which could further promote the development of cancer. Therefore, reversing the immunosuppressive effect of stress hormones on granulocytes is an urgent problem in clinical cancer treatment. Here, we selected noradrenaline (NA) as a representative stress hormone.

**Methods and results:**

After screening many traditional Chinese herbal medicine active ingredients, a promising compound, ginsenoside Rg1, attracted our attention. We verified the immunoprotective effect of ginsenoside Rg1 on granulocytes *in vitro* and *ex vivo*, and attempted to understand its potential immunoprotective mechanism. We confirmed the immunoprotective effect of ginsenoside Rg1 on granulocytes using cell and animal experiments. Cell counting kit-8 (CCK-8) and *ex vivo* experiments were performed to investigate the immunoprotective effects of ginsenoside Rg1 on the anti-cancer function of granulocytes inhibited by NA. Transcriptome sequencing analysis and qRT-PCR showed that NA elevated the mRNA expression of *ARG2*, *MMP1*, *S100A4*, and *RAPSN* in granulocytes, thereby reducing the anti-cancer function of granulocytes. In contrast, ginsenoside Rg1 downregulated the mRNA expression of *ARG2*, *MMP1*, *S100A4*, and *RAPSN*, and upregulated the mRNA expression of *LAMC2*, *DSC2*, *KRT6A*, and *FOSB*, thereby enhancing the anti-cancer function of granulocytes inhibited by NA. Transwell cell migration experiments were performed to verify that ginsenoside Rg1 significantly enhanced the migration capability of granulocytes inhibited by NA. Tumor-bearing model mice were used to verify the significant immunoprotective effects *in vivo*. Finally, CCK-8 and hematoxylin and eosin staining experiments indicated that ginsenoside Rg1 exhibited high biosafety *in vitro* and *in vivo*.

**Discussion:**

In future clinical treatments, ginsenoside Rg1 may be used as an adjuvant agent for cancer treatment to alleviate chronic stress-induced adverse events in cancer patients.

## 1 Introduction

In recent years, increasing evidence has shown that chronic stress is closely related to the development of cancer (1–7). During chronic stress, the dysregulation of the hypothalamic-pituitary-adrenal axis could further lead to the persistent abnormal secretion of stress hormones [hydrocortisone, adrenaline, and noradrenaline (NA)] in the body (8). The long-term dysregulation of neuroendocrine hormones could lead to a series of health problems, including cancer development (9–11). Thus far, numerous studies have confirmed that high stress hormone levels over a prolonged period of time can inhibit immune system function (12–14). Data showed that chronic stress could promote the proliferation and migration of tumor cells by inhibiting different functional stages of the immune system (such as antigen presentation, humoral immunity, and cellular immunity) (15–21).

Neutrophils are the primary responders to infections that display potent antimicrobial functions including phagocytosis, degranulation, and neutrophil extracelluar trap (NET) production (22, 23). Meanwhile, the results and clinical correlative evidence have revealed that neutrophil subpopulations have distinct functions under the tumour microenvironment (24, 25). Tumor-associated neutrophils (TANs) have differential states of activation: N1 antitumorigenic phenotype and N2 protumorigenic phenotype. N1 neutrophils can directly kill tumour cells through the release of ROS and RNS. They also promote T cell activation and recruitment of pro-inflammatory macrophages. N2 neutrophils promote tumor angiogenesis and inhibit NK cell function *via* the release of MMP9. In addition, they recruit anti-inflammatory macrophages and Treg cells (24, 26, 27). The results discussed above suggest that neutrophils have high heterogeneity and plasticity. Our previous study suggested that stress hormones secreted in the body during chronic stress could alter neutrophil cell function and phenotype.

In 2020, our research group reported that the high levels of stress hormones secreted in the body during chronic stress could inhibit the cancer-killing activity (CKA) of granulocytes, which could further reduce the anti-cancer function of the immune system and thus promote the occurrence and development of cancer (28). Our findings provided strong evidence to support the view that chronic stress promotes the occurrence and development of cancer.

Patients often develop chronic mental stress after the diagnosis of cancer (8, 9, 29, 30). The prolonged maintenance of elevated stress hormone levels in these patients further inhibits the CKA of granulocytes and ultimately promotes the development of cancer (8, 9). Therefore, it is necessary to develop an agent that can effectively reverse the immunosuppressive effects of stress hormones and ensure its biosafety. To address this issue, we screened a variety of bioactive components in Chinese traditional herbs during our preliminary exploration experiments. Among them, ginsenoside Rg1 attracted our attention because of its excellent immunoprotective function.

Ginsenoside Rg1 is an extract of ginseng (31). Previous reports showed that ginsenoside Rg1 can improve immunity and enhance anti-tumor activity, and exhibits broad application prospects in high-end health care and other fields (32, 33). However, no study has reported the potential application of ginsenoside Rg1 in anti-tumor therapy for patients with cancer suffering from stress. In the present study, we investigated whether ginsenoside Rg1 could enhance the CKA of stress-hormone-inhibited granulocytes. This study will also provide a new direction for the clinical application of ginsenoside Rg1 in the future.

In a previous report, we investigated the immunosuppressive effects of three stress hormones (hydrocortisone, adrenaline, and NA). Among the three stress hormones, NA exhibited the strongest immunosuppressive effect *in vivo* (28). Therefore, in this study, NA was selected as a representative stress hormone to explore the protective effect of ginsenoside Rg1 on the CKA of stress hormone-inhibited granulocytes. Here, the biosafety of ginsenoside Rg1 was investigated *via* cytotoxicity experiments; the immunoprotective effects of ginsenoside Rg1 *in vitro* were investigated using cell counting kit-8 (CCK-8) and transwell cell migration tests; the immunoprotective mechanism of ginsenoside Rg1 was investigated by transcriptome sequencing analysis and qRT-PCR; the immunoprotective function of ginsenoside Rg1 *in vivo* was verified using tumor-bearing model mice. The results of this study showed that ginsenoside Rg1 could enhance the CKA of granulocytes inhibited by NA. Ginsenoside Rg1 may find application as an immunoprotective agent to alleviate the immunosuppressive effect of chronic stress in future clinical treatments.

## 2 Materials and methods

### 2.1 Reagents

All reagents were commercially available. Ultrapure water was obtained from Millipore machines (Billerica, MA, USA). CCK-8 and Annexin V-fluorescein isothiocyanate (FITC)/propidium iodide (PI) apoptosis detection kit were purchased from Dojindo (Tokyo, Japan); Dulbecco’s Modified Eagle’s Medium (DMEM), fetal bovine serum (FBS), and phosphate-buffered saline (PBS) were purchased from Biological Industries (Kibbutz Beit Haemek, Israel); diff-quik staining solution and 4’, 6-diamidino-2-phenylindole (DAPI) were purchased from Solarbio (Beijing, China); red cells lysis bAuffer was purchased from BioGems (CA, USA); percoll was purchased from GE Healthcare Life Sciences (MA, USA); cytokeratin 19 antibody and cluster of differentiation 66 antibody were purchased from Abcam (Cambridge, UK); ginsenoside Rg1 was purchased from MACKLIN (Shanghai, China); and NA was purchased from AMQUAR (Shanghai, China).

### 2.2 Instrumentation

An HT7700 120KV electron microscope (Hitachi, Tokyo, Japan) was used for transmission electron microscopy (TEM); an ECLIPSE 80i fluorescence microscope (Nikon, Tokyo, Japan) was used for fluorescence imaging; a MULTISKAN MK3 Microplate Reader (Thermo Scientific, MA, USA) was used for cell viability tests; a BD FACSVerse (NJ, USA) was used for flow cytometry; and a Leica TCS SP5 II (Hesse, Germany) was used for confocal microscope imaging.

### 2.3 Cells

A549 and S180 cell lines were purchased from Shanghai Institute of Cell Biology (Shanghai, China).

### 2.4 Animals

In this study, 27 adult male SD rats (clean grade, weight ~160 g) and 24 adult male nude mice (SPF grade, weight ~20 g) were purchased from Shanghai SLAC Laboratory Animal Co., Ltd (Shanghai, China). Production license number: SCXK (Shanghai) 2017-0005; use license number: SYXK (Shanghai) 2017-0008. This study was approved by the Tongji University Institutional Review Board (Grant No. TJAA07221402).

### 2.5 Donors

A total of 50 healthy donors (18–25 years) from Tongji University (Shanghai, China) who consented were recruited as the study participants. Volunteers were required to fill in informed consent before donating blood. None of the participants consumed alcohol, smoked, or took any medication during the study period. This study was approved by the Tongji University Institutional Review Board (Grant No. 2019tjdx282).

### 2.6 Granulocyte isolation

From each participant, 10 mL of blood was collected by heparinized venipuncture (Yu Li, Jiangsu, China) on the day of use. Granulocytes were isolated from human whole blood *via* percoll gradient separation (28, 34).

### 2.7 CKA assay

A549 cells (8 × 10^3^ cells/well) were incubated with DMEM supplemented with 10% FBS at 37°C for 24 h. CKA was tested as described previously (28, 34). Briefly, granulocytes were added to A549 cells as effector cells: target cells (E: T) ratio of 10: 1 and incubated at 37°C for 24 h. After thorough removal of non-adherent cells, the viable target cells were determined by CCK-8 assay according to the manufacturers’ instruction. Each data point was the average of the triplicates.

### 2.8 Cytotoxicity test

#### 2.8.1 Effects of ginsenoside Rg1 on A549 cell viability

A549 cells were seeded in 96 wells plate at a concentration of 8 × 10^3^ cells/well and incubated at 37°C for 24 h. After discarding the culture medium, different concentrations of ginsenoside Rg1 (0, 0.1, 1, 10, and 100 mg/L) were added to A549 and incubated at 37°C for additional 24 h. After discarding the culture medium, CCK-8 reagent diluted with DMEM containing 10% FBS (110 µL) was added into each well and incubated at 37°C for 1–2 h. Optical density (OD) values were measured using a microtiter plate reader at 450 nm (35).

#### 2.8.2 Effects of ginsenoside Rg1 on granulocyte viability

Granulocytes were first seeded in 96-well plates at a concentration of 3 × 10^5^ cells/well. Subsequently, different concentrations of ginsenoside Rg1 (0, 0.1, 1, 10, and 100 mg/L) were added to granulocytes and incubated at 37°C for 24 h. CCK-8 reagent was then added into each well and incubated at 37°C for an additional 12 h. OD values were measured using a microtiter plate reader at 450 nm (35).

### 2.9 RNA isolation and library preparation

Total RNA was isolated from the granulocytes of healthy volunteers. The granulocytes were divided into four groups (Control group, NA group, Rg1 group, and NA + Rg1 group). Each group contained three biological duplicate samples (Control 1, Control 2, Control 3, NA 1, NA 2, NA 3, Rg1 1, Rg1 2, Rg1 3, NA + Rg1 1, NA + Rg1 2, and NA + Rg1 3). The total RNA was isolated using TRIzol reagent (Invitrogen, CA, USA). The quality and purity of the isolated RNA were assessed using a NanoDrop 2000 spectrophotometer (Thermo Scientific). The cDNA libraries were constructed using a TruSeq Stranded mRNA LT Sample Prep Kit (Illumina, CA, USA).

### 2.10 RNA sequencing and analysis of differentially expressed genes

Transcriptome sequencing and analysis were conducted by OE Biotech Co., Ltd. (Shanghai, China). The cDNA libraries were sequenced on an Illumina HiSeq X Ten platform (Illumina). Differentially expressed genes were analyzed using the DESeq (2012) R package. Kyoto Encyclopedia of Gene and Genomes (KEGG) and Gene Ontology (GO) enrichment analysis were performed using R based on the hypergeometric distribution (36). We used the STRING database to predict protein-protein interaction networks (37). The threshold of significantly differential expression was: probability (P) < 0.05 and foldchange (FC) > 2 or (FC) < 0.5.

### 2.11 qRT-PCR

Total RNA was extracted from cells using TRIzol reagent. Quantification was conducted through a two-step reaction process: reverse transcription (RT) and PCR. RT reactions were performed in a GeneAmp PCR System 9700 (Applied Biosystems, USA). Real-time PCR was performed using a LightCycler 480 II Real-time PCR Instrument (Roche, Swiss). At the end of the PCR cycles, melting curve analysis was performed to validate the specific generation of the expected PCR product. *ACTB* was used as the housekeeping gene. Relative expression quantification analysis was performed using the 2^-ΔΔCt^ method (38). The following are the details of the primers used:

*ARG2* forward: 5’- ACAACAACCTGATAGTGAATCC-3’, *ARG2* reverse: 5’- TCTGACACAGCTCTGCTAAC-3’;

*MMP1* forward: 5’-GAGGAAATCTTGCTCATGCTT-3’, *MMP1* reverse: 5’- CTCTCTGAAATTGTTGGTCCAC-3’;

*S100A4* forward: 5’- TTGGACAGCAACAGGGACAA-3’, *S100A4* reverse: 5’- AGAATTCGTTACACATCATGGC-3’;

*RAPSN* forward: 5’- TTGTGAGGTTCCACGAGT-3’, *RAPSN* reverse: 5’- GGCTGTTCTTCTCGCCTAT-3’;

*LAMC2* forward: 5’-CCCTGGGTTGAACAGTGTAT-3’, *LAMC2* reverse: 5’- AGTCTCGCTGAATCTCTCTT-3’;

*DSC2* forward: 5’- ACACGGCCCAAAACTATACCA-3’, *DSC2* reverse: 5’- TTTCCAGTGTCTCTCTCCACATA-3’;

*KRT6A* forward: 5’- CTTTCCACTGGCTCTCAAAC-3’, *KRT6A* reverse: 5’- GTCACTTGTGCTTTCATGGAT-3’;

*FOSB* forward: 5’- ACCTGACGGCTTCTCTCTTTA-3’, *FOSB* reverse: 5’- GGACAAACGAAGAAGTGTACG-3’;

*ACTB* forward: 5’- CATTCCAAATATGAGATGCGTT-3’, *ACTB* reverse: 5’- TACACGAAAGCAATGCTATCAC-3’.

### 2.12 Cell migration assay

Five healthy volunteers were recruited and 10 mL of peripheral blood was collected from each volunteer. The granulocytes were separated from whole blood *via* percoll gradient separation and divided into four groups (groups A, B, C, and E) and added into non-adherent 24-well plates. Granulocytes in group A and E were incubated at 37°C for 24 h. Those in group B were first incubated at 37°C for 12 h. Subsequently, NA (50 µg/mL) was added and the granulocytes were further incubated at 37°C for an additional 12 h. Granulocytes in group C were first incubated with ginsenoside Rg1 (100 mg/L) at 37°C for 12 h. Subsequently, NA (50 µg/mL) was added and the granulocytes were further incubated at 37°C for an additional 12 h. The granulocytes in the above four groups were then collected and centrifuged at 1000 rpm for 5 min. After discarding the culture medium, granulocytes were dispersed in DMEM and counted using Countstar (ALIT Life Science, Shanghai, China). Transwell chambers were placed in a 24-well plate inoculated with or without A549 cells (600 µL, 2.5 × 10^5^ cells/well). Granulocyte suspension (100 µL, E: T = 10: 1) was added to the upper chambers and incubated at 37°C for 3 h (A549 cells were pre-inoculated in the lower chambers of group A, B, and C; no cells were pre-inoculated in the lower chambers of group E). Subsequently, cells in the upper chambers were wiped off with cotton swabs. Cells in the lower chambers were digested with trypsin and resuspended in PBS solution. Cells were counted using Countstar (cell numbers in the lower chambers of groups A, B, C, and E were recorded as A, B, C, and E, respectively). The number of A549 cells inoculated was recorded as D. The chemotactic index of granulocytes in group A = (A - D)/E; chemotactic index of granulocytes in group B = (B - D)/E; chemotactic index of granulocytes in group C = (C - D)/E (39).

### 2.13 *In vivo* immunoprotective evaluation of ginsenoside Rg1

On day 0, 24 healthy nude mice were randomly divided into four groups: Control group, NA group, NA + Rg1 group, and Rg1 group. On day 1, mice in the Control group were intraperitoneally (i.p.) injected with 100 µL PBS per day for 24 consecutive days; mice in the NA group were i.p. injected with NA (100 µL, 2 mg/kg) per day for 24 consecutive days; mice in NA + Rg1 group were i.p. injected with NA (100 µL, 2 mg/kg) and ginsenoside Rg1 (100 µL, 50 mg/kg) per day for 24 consecutive days; mice in Rg1 group were i.p. injected with ginsenoside Rg1 (100 µL, 50 mg/kg) per day for 24 consecutive days. On day 8, ascites tumor S180 cells (500 µL, 2 × 10^6^ cells/mL) were inoculated into the abdominal cavity of the mice in the 4 groups. The body weight, abdominal circumference, average food consumption, and survival rate were recorded daily for 24 days.

### 2.14 Statistic analysis

Three biological replicates were performed for all experiments in this study, unless otherwise indicated. Statistical analyses were performed using GraphPad Prism software, version 7.01 (GraphPad Software, San Diego, CA, USA). One-way analysis of variance with Dunnett post-test was used to analyze the differences between multiple groups. Two-tailed Student’s t-test was used to analyze the differences between the two groups. Log-rank test was used to analyze the survival curve. Statistical significance was set at a P value of less than 0.05, 0.01, or 0.001, indicated by *, **, and ***, respectively.

## 3 Results

### 3.1 Granulocytes from the peripheral blood of healthy humans exhibited anticancer function

**Figure 1:**
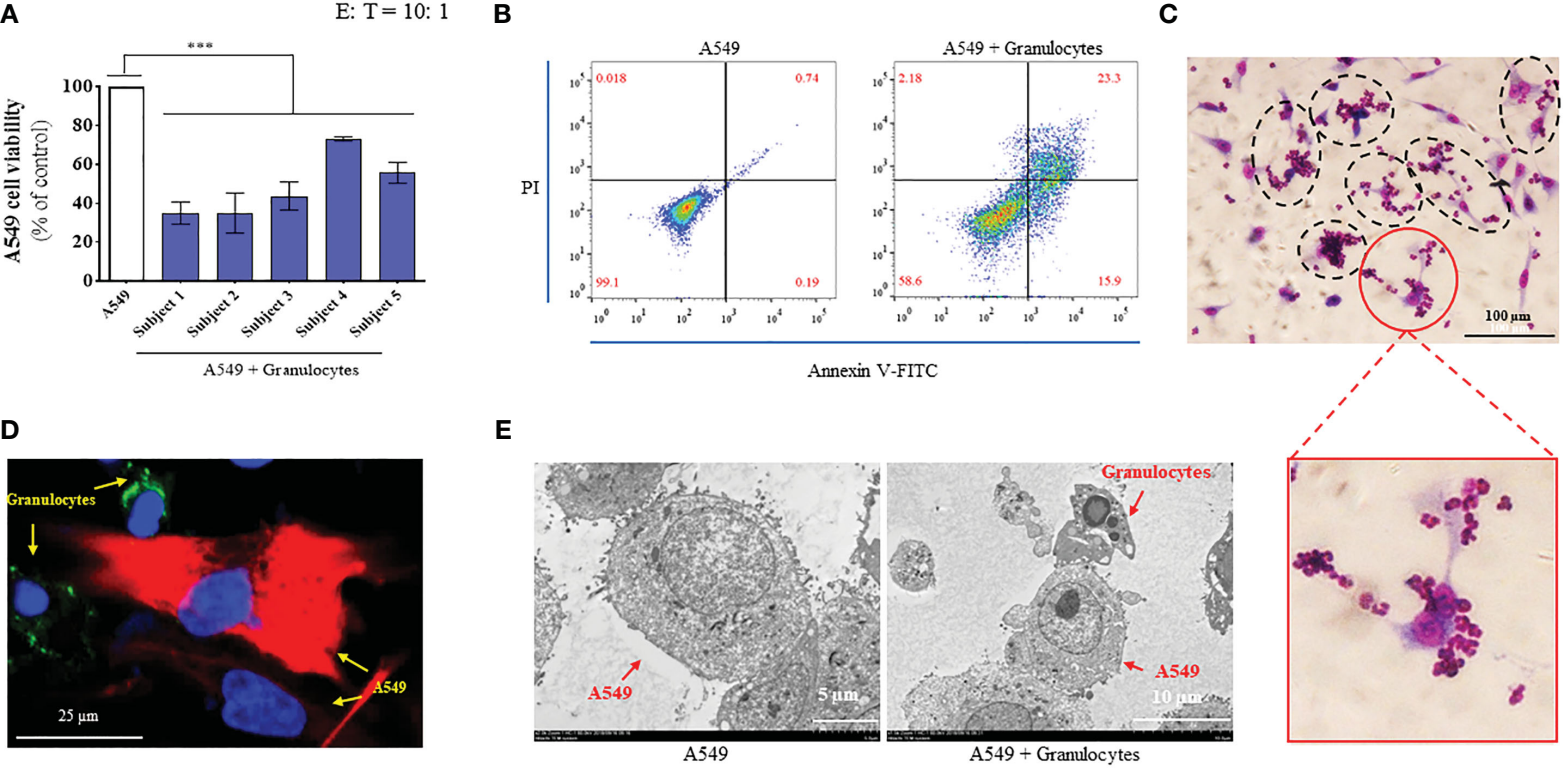
Granulocytes collected from the peripheral blood of healthy humans exhibited significant anticancer function. **(A)** Cancer-killing activity of granulocytes from 5 healthy volunteers tested using cell counting kit-8 (CCK-8) reagent (n = 3; mean ± SD; one-way analysis of variance with Dunnett post-test; ***, P < 0.001). **(B)** Cancer-killing efficiency of human granulocytes against A549 tested by flow cytometry (Annexin V-FITC/PI). **(C)** Images of granulocytes attacking A549 by diff-quik staining (magnification: 20 ×). **(D)** Confocal microscope image of granulocytes attacking A549 (magnification: 60 ×). Here, A549 cells were marked by CK19 (red), granulocyte cells were marked by CD66 (green), and the nuclei were marked by DAPI (blue). **(E)** Transmission electron microscopy (TEM) images of A549 (left, magnification: 2000 ×) and granulocyte attacking A549 (right, magnification: 1500 ×).

We recruited five healthy volunteers and collected 10 mL of peripheral blood from each volunteer. We separated granulocytes from whole blood *via* percoll gradient separation and tested the CKA of granulocytes. Firstly, we tested the cancer-killing efficiency of granulocytes from five healthy volunteers by CCK-8 (target cells: A549; E: T = 10: 1) (28). The results showed that the granulocytes from the five healthy volunteers exhibited significant cancer-killing efficiency in the range of 35–73%, which was consistent with the result of our previous report (**Figure 1A**) (28). Next, we further verified the anti-cancer function of granulocytes by flow cytometry. Granulocytes were added to A549 cells (E: T = 10: 1) and incubated at 37°C for 24 h. After thoroughly discarding non-adherent cells and washing A549 with PBS three times, A549 cells were harvested and stained with Annexin V-FITC and PI. In the detection results, the cells divided into the first quadrant were mechanically damaged cells, those in the second quadrant were non-viable apoptotic cells or cells that experienced secondary cell death, those in the third quadrant were normal living cells, and those in the fourth quadrant were viable apoptotic cells (40). The flow cytometry results showed that 99.1% of the A549 group were normal living cells, 0.018% were mechanically damaged cells, 0.19% were viable apoptotic cells, and 0.74% were non-viable apoptotic cells or cells that experienced secondary cell death. While 58.6% of the A549 + granulocyte group were normal living cells, 2.18% were mechanically damaged cells, 15.9% were viable apoptotic cells, and 23.3% were non-viable apoptotic cells or cells that experienced secondary cell death. Compared with the A549 group, the percentage of normal living A549 cells decreased from 99.1% to 58.6% in the A549 + granulocytes group. Moreover, the percentage of apoptotic cells (including viable apoptotic cells and non-viable apoptotic cells) in the A549 + granulocytes group increased from 0.93% to 39.2% (**Figure 1B**). The above results indicated that the A549 cells were mainly killed by granulocytes *via* the apoptosis pathway. Furthermore, diff-quik staining, confocal microscopy, and TEM images showed that when granulocytes attacked A549 cells, the A549 cells were surrounded by granulocytes to form a “rosette” structure (**Figures 1C-E**), which was consistent with our previous report (41). Simultaneously, A549 cells exhibited apoptotic characteristics (**Figure 1E**), which was consistent with the results of flow cytometry.

### 3.2 Ginsenoside Rg1 enhanced the anti-cancer function of granulocytes inhibited by NA *in vitro*

Our previous study showed that stress hormones (hydrocortisone, adrenaline, and NA) were secreted under conditions of stress, which could inhibit the function of the immune system and reduce the CKA efficiency of granulocytes (28). Additionally, among the three stress hormones mentioned above, NA may be the strongest inhibitor of CKA of granulocytes *in vivo* (28). This raised the question as to whether ginsenoside Rg1 could enhance the anti-cancer function of stress hormone-inhibited granulocytes. The present study focused on NA.

**Figure 2:**
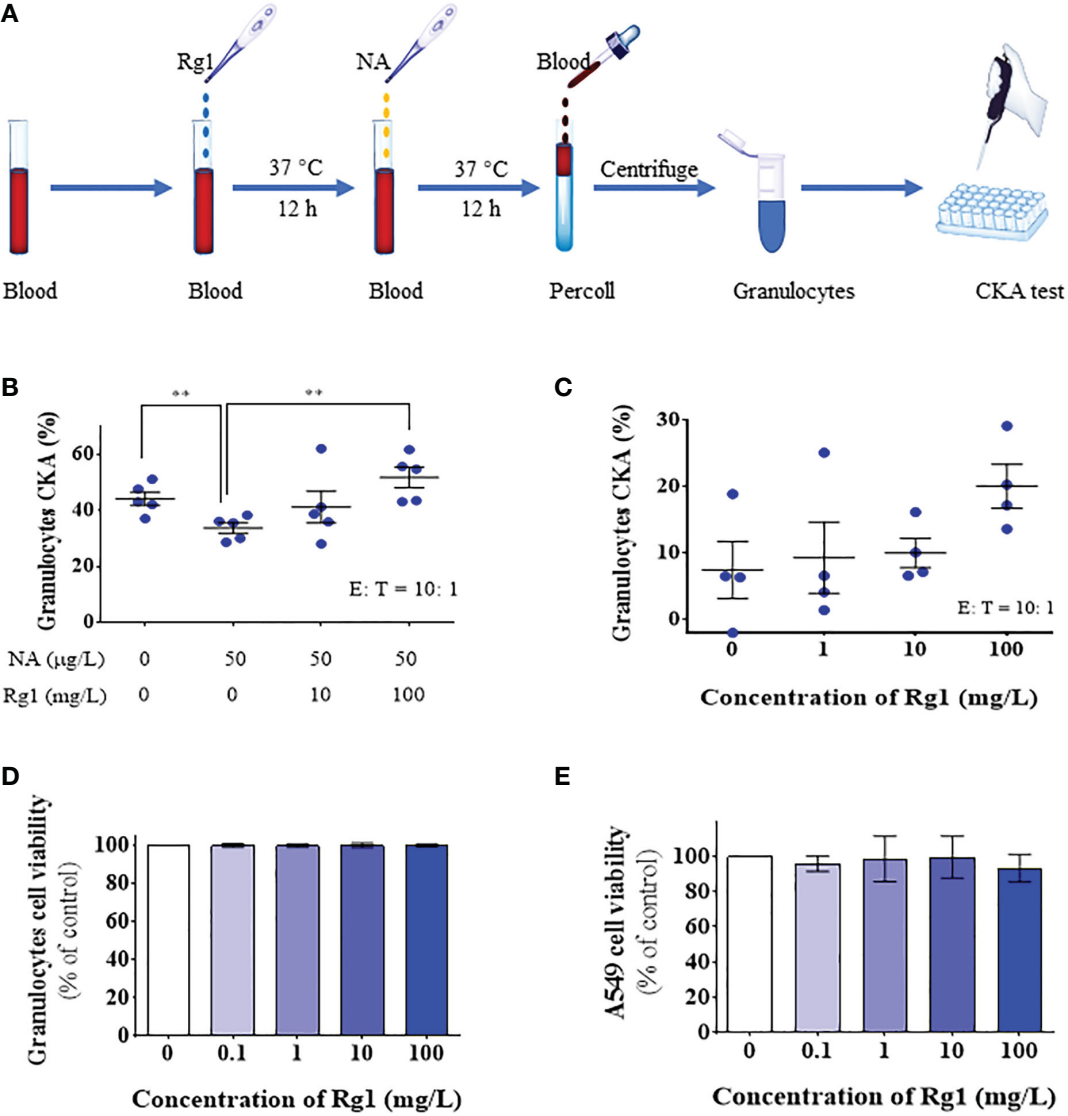
*In vitro*, ginsenoside Rg1 could enhance the anti-cancer function of granulocytes inhibited by noradrenaline (NA). **(A)** Detection of the effects of ginsenoside Rg1 on the anti-cancer function of granulocytes inhibited by NA. **(B)** The effects of ginsenoside Rg1 on granulocyte cancer-killing activity **(CKA)** immunosuppressed by NA at different concentrations (n = 5; mean ± SD; two-tailed student’s t-test; **, P < 0.01). **(C)** The effects of ginsenoside Rg1 on granulocyte CKA at different concentrations. **(D)** The effects of ginsenoside Rg1 on granulocyte cell viability at different concentrations. **(E)** The effects of ginsenoside Rg1 on A549 cell viability at different concentrations.

First, we explored the cytotoxicity of ginsenoside Rg1. Ginsenoside Rg1 was added to human granulocytes or A549 human lung cancer cells at concentrations of 0, 0.1, 1, 10, and 100 mg/L and incubated at 37°C for 24 h. Cell survival rate was measured using CCK-8. The experiment results are shown in **Figures 2D-E**. Data showed that 0–100 mg/L ginsenoside Rg1 exhibited no significant inhibitory effect on the survival rate of human granulocytes and A549 cells, indicating that ginsenoside Rg1 was biologically safe. These results were consistent with those of previous reports (42).

Subsequently, different concentrations of ginsenoside Rg1 (0, 10, and 100 mg/L) were added to human whole blood and incubated at 37°C for 12 h. NA (50 µg/L) was then added to the whole blood and incubated at 37°C for an additional 12 h. After isolating granulocytes from the whole blood, cancer cell viability was measured using CCK-8 (**Figure 2A**). The experiment results are shown in **Figure 2B**. The data showed that 1) NA inhibited the CKA of human granulocytes, which is consistent with the results of our previous study (28); 2) ginsenoside Rg1 significantly enhanced the cancer-killing efficiency of granulocytes immunosuppressed by NA; 3) ginsenoside Rg1 exhibited dose-dependent immunoprotective effects. At the concentration range explored in this study, the CKA of granulocytes increased with increasing Rg1 concentration.

Finally, we investigated the effects of ginsenoside Rg1 on the CKA of granulocytes in healthy humans. In this experiment, different concentrations of ginsenoside Rg1 were added to human whole blood and incubated at 37°C. After 24 h, the CKA of human granulocytes was measured using CCK-8. The results were shown in **Figure 2C**. The data showed that 1) ginsenoside Rg1 improved the CKA of human granulocytes to a certain extent, but the improvement showed no statistical significance; 2) ginsenoside Rg1 showed a concentration-dependent enhancement effect on the CKA of human granulocytes. At the concentration range explored in this study, the CKA of granulocytes increased with increasing Rg1 concentration.

### 3.3 Ginsenoside Rg1 could enhance the cancer-killing efficiency of granulocytes immunosuppressed by NA in healthy rats *ex vivo*

**Figure 3:**
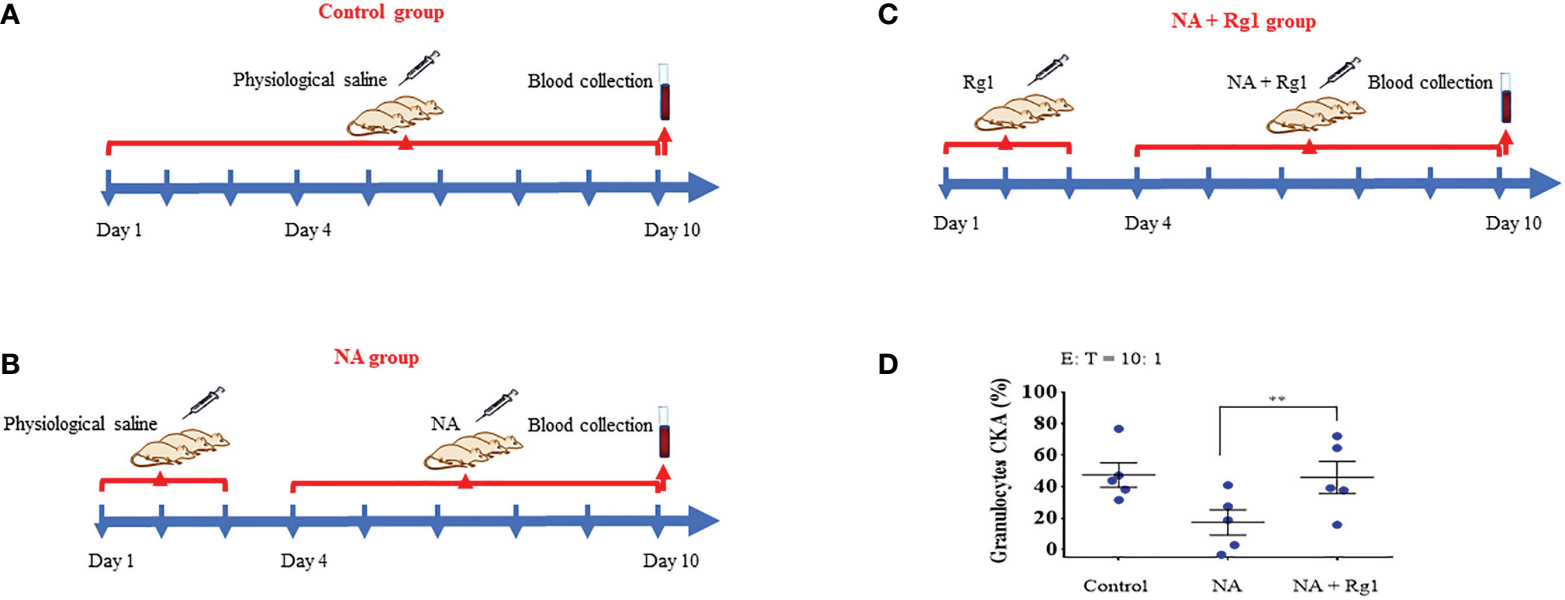
In the *ex vivo* test, ginsenoside Rg1 could improve the cancer-killing efficiency of rats granulocytes immunosuppressed by noradrenaline (NA). **(A - C)** Detection of the effect of ginsenoside Rg1 on the cancer-killing efficiency of granulocytes in rats immunosuppressed by NA. **(D)** Effect of ginsenoside Rg1 (20 mg/kg) on the cancer-killing efficiency of granulocytes immunosuppressed by NA in rats (n = 5; mean ± SD; two-tailed student’s t-test; **, P < 0.01).

We investigated the immunoprotective effects of ginsenoside Rg1 in an *ex vivo* test. In this experiment, rats in the Control group were i.p. injected with 1.5 mL saline per day for 10 days; rats in the NA group were first i.p. injected with 1.5 mL saline per day for 3 days, then i.p. injected with 50 µg/kg NA per day for 7 days; rats in the NA + Rg1 group were first i.p. injected with 20 mg/kg ginsenoside Rg1 per day for 3 days, then i.p. injected with 20 mg/kg ginsenoside Rg1 and NA (50 µg/kg) per day for 7 days. On the 10^th^ day, blood was collected 2 h after injection, and the CKA of granulocytes was detected (**Figures 3A-C**). The results are shown in **Figure 3D**. The results showed that 1) NA inhibited the CKA of granulocytes in healthy rats to a certain extent, but the inhibitory effect was not statistically significant; 2) ginsenoside Rg1 significantly enhanced the cancer-killing efficiency of granulocytes immunosuppressed by NA in healthy rats, which was consistent with the *in vitro* results.

Furthermore, we investigated the effects of ginsenoside Rg1 on the CKA of granulocytes in healthy rats. In this experiment, rats in the Control group were i.p. injected with 1.5 mL saline per day for 10 days; rats in the Rg1 group were i.p. injected with 20 mg/kg ginsenoside Rg1 per day for 10 days. Blood was collected on the 10^th^ day and the CKA of granulocytes was tested using CCK-8 (**Figure S1A–B**). The results showed that ginsenoside Rg1 had no significant effect on the CKA of the granulocytes of healthy rats, indicating that ginsenoside Rg1 has no significant toxic effect on the CKA of granulocytes in healthy rats (**Figure S1C**).

### 3.4 Transcriptome analysis

**Figure 4:**
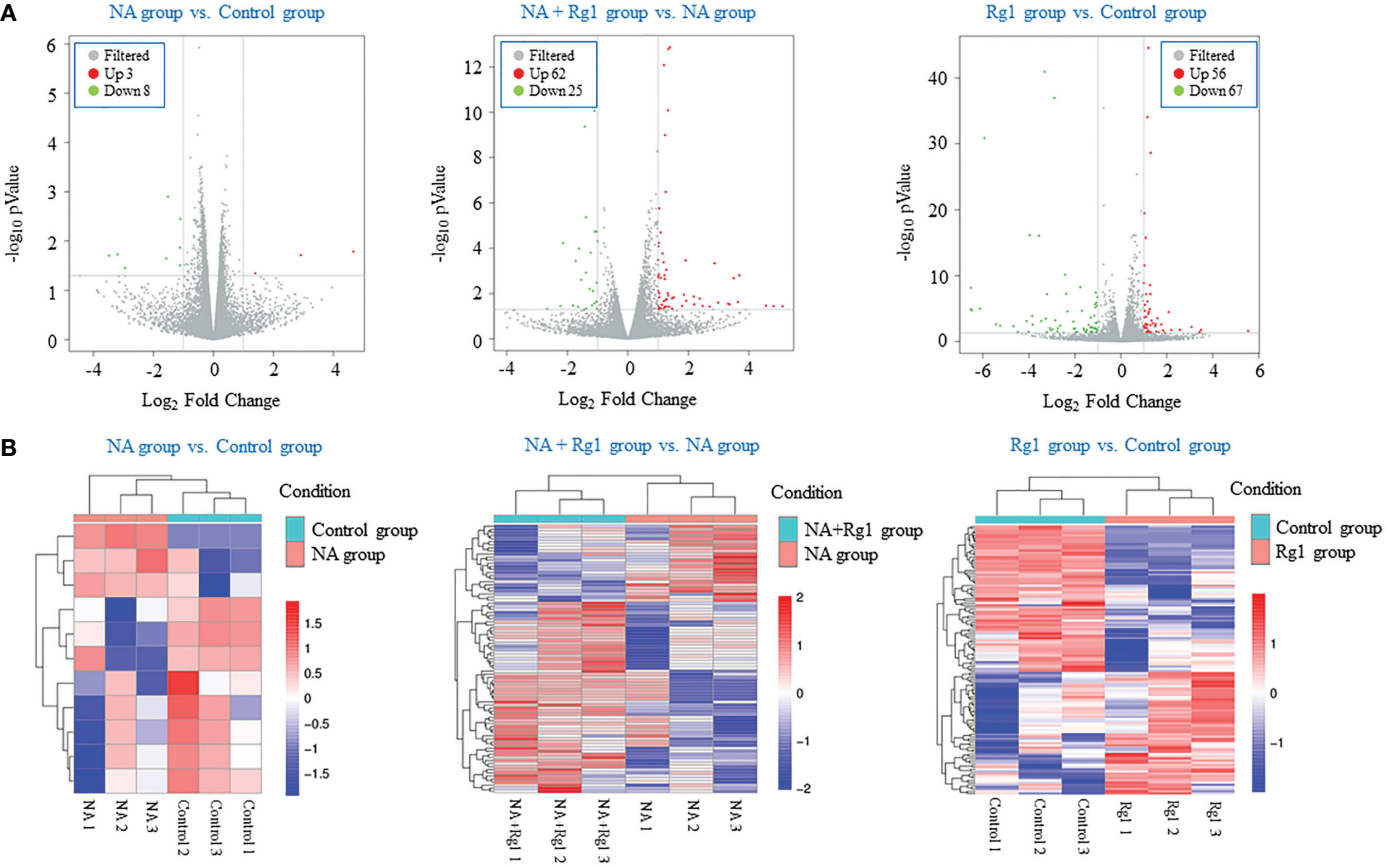
Transcriptome analysis of the effects of noradrenaline (NA) and ginsenoside Rg1 on the gene expression of granulocyte from healthy humans. **(A)** Volcano plot of data comparison between different groups. Every dot represents a differentially expressed gene. Gray represents no significant difference in gene expression between the two groups. Red represents significantly upregulated gene expression. Green represents significantly downregulated gene expression. **(B)** Clustering analysis of differentially expressed genes between different groups. Red represents upregulated genes. Blue represents downregulated genes. The depth of different colors represents different levels of gene expression.

We performed transcriptome analysis to explore the immunoprotective mechanism of ginsenoside Rg1 on the anti-cancer functions of granulocytes inhibited by NA (43). In this study, we performed a comparative RNA-seq analysis on four groups (NA group vs Control group, NA + Rg1 group vs NA group, and Rg1 group vs Control group) including 12 samples (Control 1, Control 2, Control 3, NA 1, NA 2, NA 3, Rg1 1, Rg1 2, Rg1 3, NA + Rg1 1, NA + Rg1 2, and NA + Rg1 3). Here, granulocytes in the Control group were not treated. Granulocytes in the NA group were treated with NA (50 µg/L) at 37°C for 12 h. Granulocytes in NA + Rg1 group were first treated with ginsenoside Rg1 (100 mg/L) for 12 h, then treated with ginsenoside Rg1 (100 mg/L) and NA (50 µg/L) for an additional 12 h at 37°C. Granulocytes in the Rg1 group were treated with ginsenoside Rg1 (100 mg/L) at 37°C for 24 h. The analysis results are shown in **Figure 4**. The screening conditions for differentially expressed genes are as follows: FC ≥ 2 and P < 0.05. **Figure 4A** shows the volcano plot of data comparison between different groups, which demonstrated the overall distribution of differentially expressed genes. In the NA group vs Control group, a total of 11 differentially expressed genes, namely 3 upregulated and 8 downregulated genes, were found. In the NA + Rg1 group vs NA group, a total of 87 differentially expressed genes, namely 62 upregulated and 25 downregulated genes, were found. In the Rg1 group vs Control group, a total of 123 differentially expressed genes, namely 56 upregulated and 67 downregulated genes, were found. **Figure 4B** shows the cluster analysis of differentially expressed genes between different groups. The result shows that the trend of differentially expressed genes was consistent among different samples in the same group, while there were significant differences among different treatment groups.

### 3.5 Predicted mechanism whereby ginsenoside Rg1 enhances the anti-cancer function of NA-inhibited granulocytes

**Figure 5:**
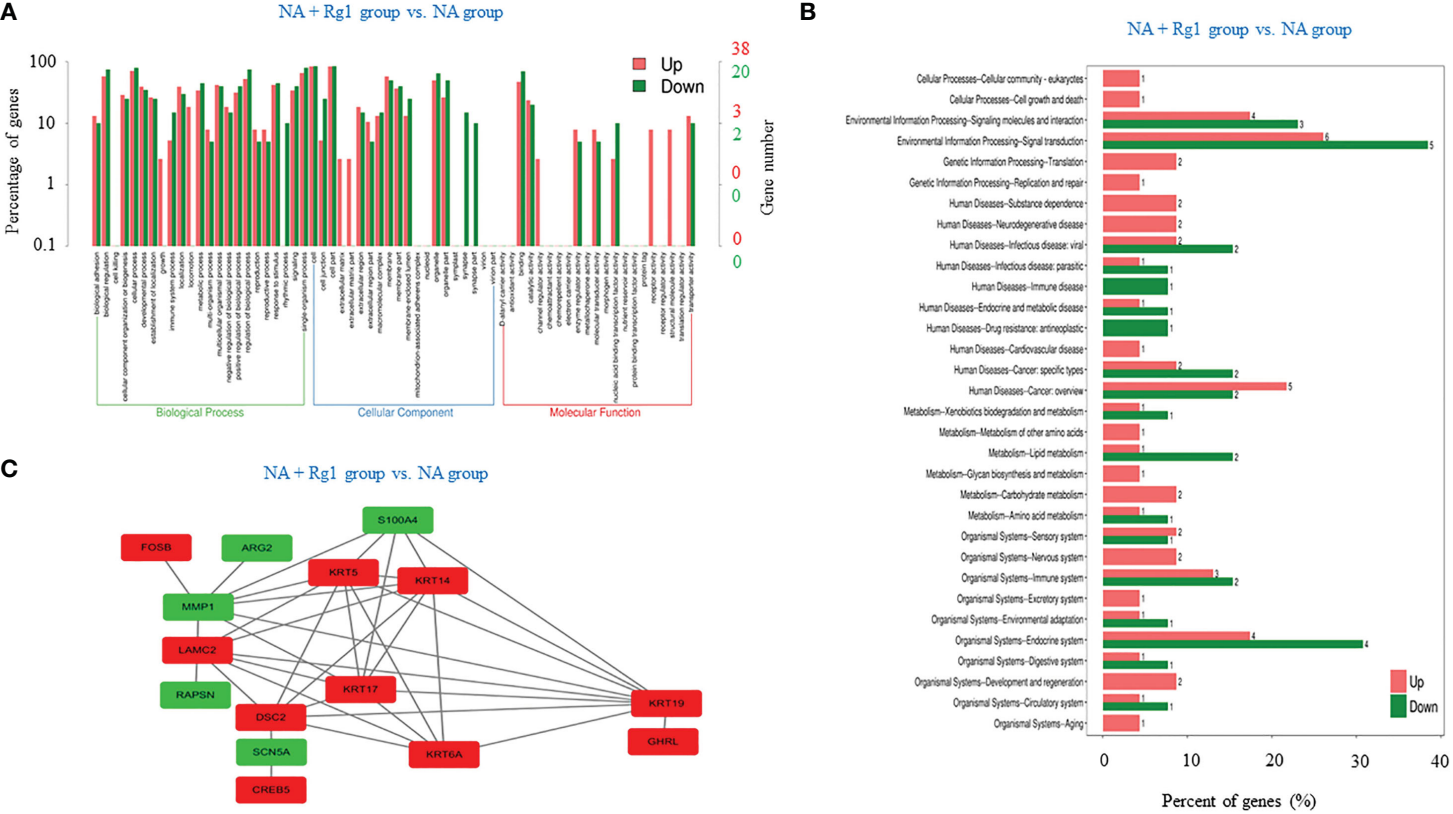
Correlation analysis of differentially expressed genes between the noradrenaline (NA) + Rg1 group and NA group. **(A)** Gene ontology (GO) enrichment of differentially expressed genes between the NA + Rg1 group and NA group. Three GO categories include biological process, cell component, and molecular function. Red represented upregulated genes, and green represented downregulated genes. **(B)** Kyoto Encyclopedia of Gene and Genomes **(KEGG)** analysis of differentially expressed genes between the NA + Rg1 group and NA group. Red represented upregulated genes, and green represented downregulated genes. **(C)** Predicted protein-protein interaction networks. Protein interaction networks that predicted 15 differential genes using the STRING database. Red represented upregulated genes, and green represented downregulated genes.

We focused on the analysis of differentially expressed genes between the NA + Rg1 group and NA group to discuss the possible molecular mechanisms by which ginsenoside Rg1 enhanced the anti-cancer function of granulocytes inhibited by NA. First, we performed GO enrichment analysis of differentially expressed genes between the NA + Rg1 group and the NA group (**Figure 5A**). The analysis showed a total of 87 differentially expressed genes between the NA + Rg1 group and the NA group, which could be divided into three main GO categories: biological processes, cellular components, and molecular functions. Among them, the 62 upregulated differentially expressed genes were assigned to 43 GO terms, including 21 biological processes, 13 cellular components, and 9 molecular functions. The 25 downregulated differentially expressed genes were assigned to 39 GO terms, including 20 biological processes, 13 cellular components, and 6 molecular functions. Moreover, we performed a signaling pathway analysis of differentially expressed genes between the NA + Rg1 group and the NA group using the KEGG database (**Figure 5B**). The result showed that a total of 56 upregulated and 32 downregulated differentially expressed genes between the NA + Rg1 group and the NA group were categorized into known KEGG pathways. Among the 56 upregulated genes, 2 genes were distributed in cellular processes, 10 genes in environmental information processing, 3 genes in genetic information processing, 16 genes in human diseases, 7 genes in metabolism, and 18 genes in organic systems. Among the 32 downregulated genes, 8 genes were distributed in environmental information processing, 10 genes in human diseases, 4 genes in metabolism, and 10 genes in organismal systems. In our previous studies, the granulocyte-mediated killing of cancer cells was roughly divided into 3 stages: chemotaxis, recognition, and killing (28, 44). Therefore, we considered that factors related to cell migration, cell adhesion, cytoskeleton composition, cell proliferation and apoptosis, and immune response may all be associated with the immunoprotective effect of ginsenoside Rg1 on granulocytes inhibited by NA. Therefore, among the 87 differentially expressed genes between the NA + Rg1 group and the NA group, we screened 15 genes that might be related to the immunoprotective effects of ginsenoside Rg1 on granulocytes inhibited by NA (**Table 1**). Furthermore, we predicted the protein-protein interaction networks for these 15 genes using the STRING database (**Figure 5C**). The prediction results showed a possible association among the 15 genes.

**Table 1:** 15 genes that may be associated with increased granulocytes cancer-killing efficiency (NA + Rg1 group vs NA group).

Gene symbol	Description	Function	Fold change (log_2_)
*LAMC2*	Laminin subunit gamma 2	Cell adhesion, positive regulation of cell migration	2.86
*DSC2*	Desmocollin 2	Cell adhesion	1.06
*KRT5*	Keratin 5	Cytoskeleton organization	1.28
*KRT6A*	Keratin 6A	Cytoskeleton organization	4.81
*KRT14*	Keratin 14	Structural constituent of cytoskeleton	3.50
*KRT17*	Keratin 17	Enables structural molecule activity, intermediate filament organization, positive regulation of cell growth, positive regulation of translation	2.38
*KRT19*	Keratin 19	Structural constituent of cytoskeleton	5.11
*GHRL*	Ghrelin and obestatin prepropeptide	Actin polymerization or depolymerization, negative regulation of apoptotic process	1.33
*CREB5*	cAMP responsive element binding protein 5	Enables cAMP response element binding	1.00
*FOSB*	FosB proto-oncogene, AP-1 transcription factor subunit	Regulators of cell proliferation, differentiation, and transformation	1.02
*SCN5A*	Sodium voltage-gated channel alpha subunit 5	Tetrodotoxin-resistant voltage-gated sodium channel subunit	-2.22
*RAPSN*	Receptor associated protein of the synapse	Enables acetylcholine receptor binding, enables ionotropic glutamate receptor binding, enables metal ion binding, enables protein-membrane adaptor activity	-1.09
*MMP1*	Matrix metallopeptidase 1	Breakdown of extracellular matrix in normal physiological processes, as well as in disease processes	-1.54
*S100A4*	S100 calcium binding protein A4	This protein may function in motility, invasion, and tubulin polymerization.	-1.10
*ARG2*	Arginase 2	Enables arginase activity, involved in adaptive immune response, involved in innate immune response	-1.61

### 3.6 Verification of the immunoprotective mechanism of ginsenoside Rg1

**Figure 6:**
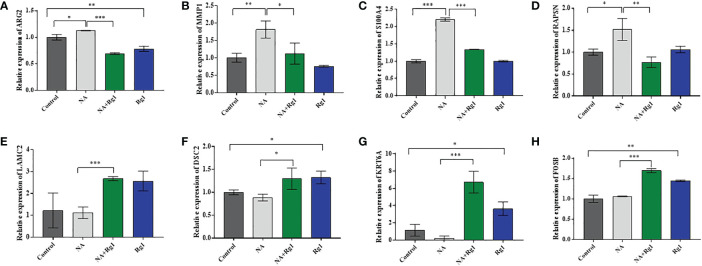
mRNA expression of *ARG2* **(A)**, *MMP1* **(B)**, *S100A4* **(C)**, *RAPSN* **(D)**, *LAMC2* **(E)**, *DSC2* **(F)**, *KRT6A* **(G)**, and *FOSB* **(H)** (n = 3; mean ± SD; two-tailed student’s *t*-test; *, *P* < 0.05; **, *P* < 0.01; ***, *P* < 0.001).

**Figure 7:**
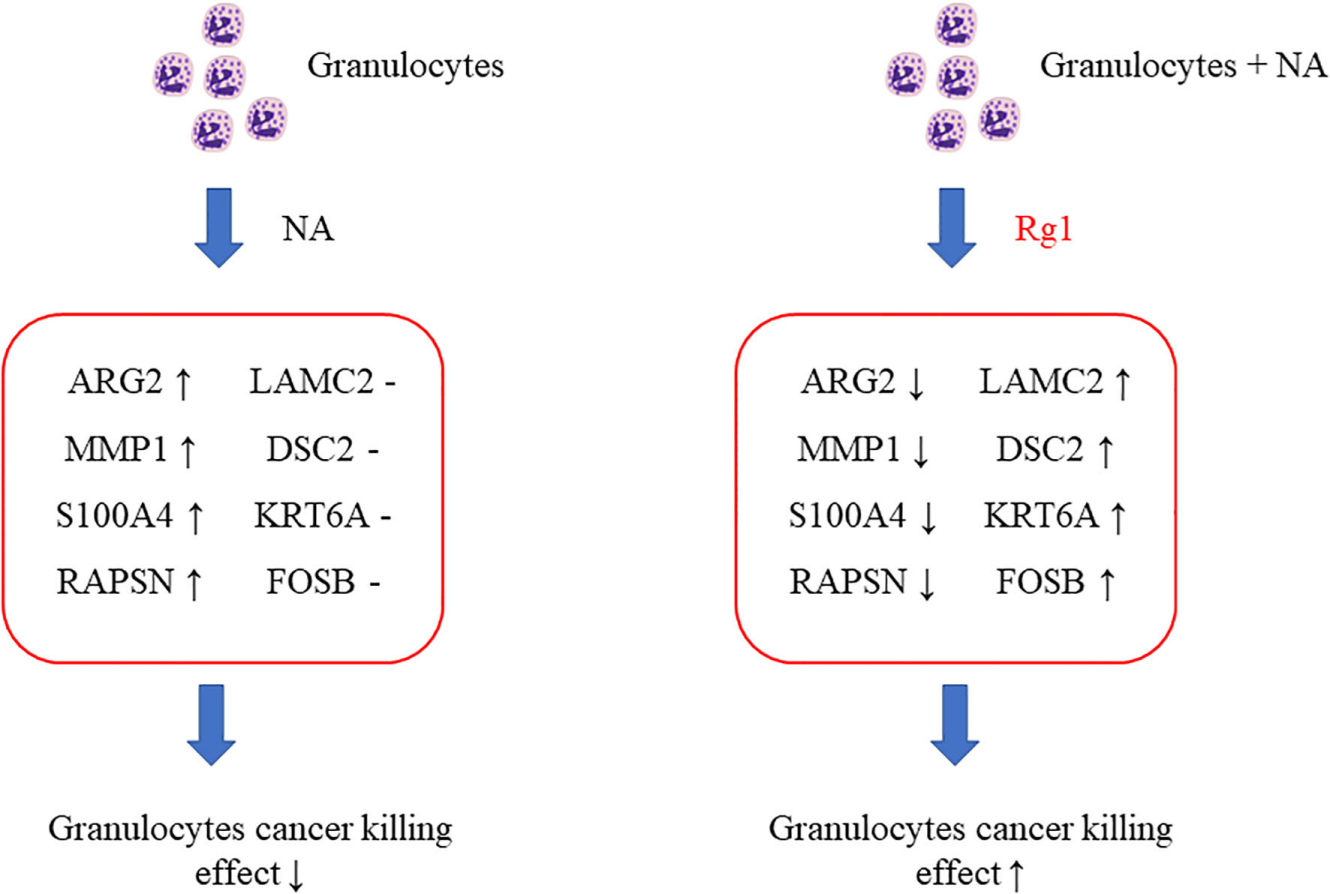
Schematic diagram about the mechanism of ginsenoside Rg1 promoting the anti-cancer function of granulocytes immunosuppressed by noradrenaline (NA).

Among the 15 genes screened above (**Table 1**), we selected 8 genes of interest (*ARG2*, *MMP1*, *S100A4*, *RAPSN*, *LAMC2*, *DSC2*, *KRT6A*, and *FOSB*), and measured the mRNA expression of these genes using qRT-PCR. The results showed that, compared with the Control group, the mRNA expression levels of *ARG2*, *MMP1*, *S100A4*, and *RAPSN* were significantly elevated in NA group. However, the mRNA expression level of these four genes was significantly reduced in the NA + Rg1 group (**Figures 6A-D**). These results were consistent with the results of transcriptome analysis (**Table 1**). In addition, compared with the Control group, the mRNA expression levels of *LAMC2*, *DSC2*, *KRT6A*, and *FOSB* showed no statistically significant difference in the NA group. Compared with the NA group, the mRNA expression level of these four genes was significantly elevated in the NA + Rg1 group (**Figures 6E-H**). These results were consistent with the results of transcriptome analysis (**Table 1**). These results suggested that NA inhibited granulocyte CKA by elevating the expression of *ARG2*, *MMP1*, *S100A4*, and *RAPSN*. Moreover, ginsenoside Rg1 could enhance granulocyte CKA (which was inhibited by NA) by inhibiting the expression of *ARG2, MMP1, S100A4*, and *RAPSN* while simultaneously elevating the expression of *LAMC2, DSC2, KRT6A*, and *FOSB* (**Figure 7**).

Previous research reported that the high expression of *ARG2* was related to immunosuppressive microenvironments (45, 46); The high expression of *MMPs* was associated with N2 tumor-associated neutrophils (47); and the overexpression of *S100A4* would promote the metastasis, invasion, and angiogenesis of cancer cells, which are related to poor prognosis in patients with cancer (48–50). The decreased methylation of *RAPSN* would upregulate the function of *RAPSN* and further accelerate downstream pathways, which was positively correlated with the development of lung cancer (51, 52). The above analysis indicated that the mRNA expression of *ARG2, MMP1, S100A4*, and *RAPSN* in neutrophils was significantly increased after the regulation of NA. These changes could inhibit the anti-cancer function of granulocytes and promote the development of cancer.

Additionally, *LAMC2* promotes the chemotactic function of granulocytes (53, 54); *DSC2* correlates positively with adhesion, migration, and infiltration of granulocytes (55, 56); and *KRT6A* protein inhibits the proliferation, migration and invasion abilities of lung cancer cells. The high expression of *KRT6A* protein is related to good prognosis in patients with lung adenocarcinoma (57); *FOSB* protein plays an anti-tumor role in lung cancer (58). These results indicated that ginsenoside Rg1 significantly inhibited the mRNA expression of *ARG2, MMP1, S100A4*, and *RAPSN* in granulocytes inhibited by NA and, simultaneously, significantly elevated the mRNA expression of *LAMC2, DSC2, KRT6A*, and *FOSB*. These changes could enhance the anti-cancer function of granulocytes and inhibit cancer cell development and progression.

### 3.7 Verification of the immunoprotective effects of ginsenoside Rg1 on granulocytes *in vitro*

We validated the *in vitro* immunoprotective effects of ginsenoside Rg1 using cell migration assays. The data showed that NA inhibited the migration capability of granulocytes and that ginsenoside Rg1 significantly enhanced the migration capability of granulocytes inhibited by NA (**Figure S2**). The data above was consistent with the changing trend of *LAMC2* and *DSC2* in **Figures 6**–**7**.

### 3.8 Verification of the immunoprotective effects of ginsenoside Rg1 on granulocytes *in vivo*

**Figure 8:**
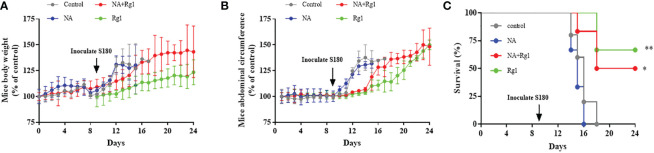
Immunoprotective effects of ginsenoside Rg1 *in vivo*. **(A)** Data of mouse body weight. **(B)** Data of mice abdominal circumference. **(C)** Data of survival rate. (n ≥ 5; log-rank test; *, *P* < 0.01; **, *P* < 0.001).

We validated the *in vivo* immunoprotective effects of ginsenoside Rg1 in tumor-bearing model mice. On day 0, healthy nude mice were randomly divided into 4 groups: Control group, NA group, NA + Rg1 group, and Rg1 group. From day 1 to day 24, mice in the Control group were i.p. injected with PBS (100 µL) per day; mice in NA group were i.p. injected with NA (2 mg/kg, 100 µL) per day; mice in NA + Rg1 group were i.p. injected with NA (2 mg/kg, 100 µL) and ginsenoside Rg1 (50 mg/kg, 100 µL) per day; mice in Rg1 group were i.p. injected with ginsenoside Rg1 (50 mg/kg, 100 µL) per day. On day 9, we inoculated S180 cells into the abdominal cavity of healthy nude mice to establish the ascites tumor model mice. For 24 days, the body weight, abdominal circumference, average food consumption, and survival rate of the mice were recorded daily. The results are shown in **Figure 8**.

The data showed that there was no significant difference in mouse body weight among the four experimental groups before inoculation with S180 cells. After inoculation with S180 cells, the mouse body weight in the Control group and NA group increased rapidly; the mouse body weight in the NA + Rg1 group increased at a slower rate than that in the Control group and NA group; while the mouse body weight in the Rg1 group had the slowest rate of increase among the four groups (**Figure 8A**). The abdominal circumference data showed that there was no significant difference among the four experimental groups before inoculation with S180 cells. After inoculation with S180 cells, the mouse abdominal circumference in the Control group and NA group increased rapidly; mouse abdominal circumference in the NA + Rg1 group increased at a slower rate than that in the Control group and NA group, while the mouse abdominal circumference in the Rg1 group had the slowest increase (**Figure 8B**). Before inoculation with S180 cells, the mice average food consumption in the NA group and the NA + Rg1 group decreased rapidly; while the mouse average food consumption in the Control group and Rg1 group did not change significantly. After inoculation with S180 cells, the mouse average food consumption in the Control group and NA group decreased rapidly; while the mouse average food consumption in the NA + Rg1 group and Rg1 group decreased relatively slowly (**Figure S3**). In addition, before inoculation with S180 cells, the mouse survival rate in the four groups was 100%. After inoculation with S180 cells, mice in the NA group died the fastest, with a median survival time of 15 days. On day 16, all mice in the NA group had died; mice in the Control group also died quickly, with a median survival time of 16 days. On day 18, mice in the Control group were all dead; in contrast, mice in the NA + Rg1 group died slower than those in the Control group and NA group, with a median survival time of 18 days. On day 24, 50% of the mice were still alive; mice in the Rg1 group died the slowest, with a median survival time longer than 24 days. On day 24, 67% of the mice in this group still survived (**Figure 8C**). Data above indicated that ginsenoside Rg1 showed significant immunoprotective effects *in vivo*, which prolonged the survival time and slowed the growth of ascites tumors in tumor-bearing mice immunosuppressed by NA.

### 3.9 Toxicity test of ginsenoside Rg1 *in vivo*

**Figure 9:**
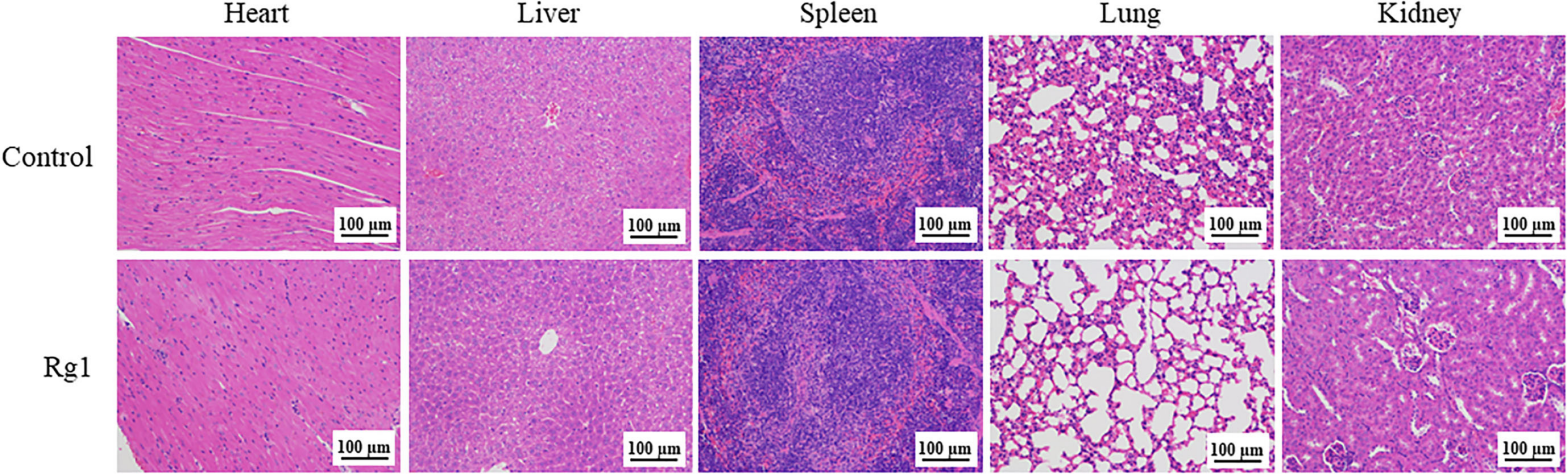
Healthy nude mice were intraperitoneally (i.p.) injected with phosphate-buffered saline (PBS; 100 µL, Control group) and ginsenoside Rg1 (50 mg/kg, 100 µL, Rg1 group), respectively. After 24 h, the heart, liver, spleen, lung, and kidney were stained with hematoxylin and eosin (H&E) staining reagents.

We assessed the *in vivo* toxicity of ginsenoside Rg1 by hematoxylin and eosin (H&E) staining. In this test, ginsenoside Rg1 (50 mg/kg, 100 µL, Rg1 group) and PBS (100 µL, Control group) were i.p. injected into healthy nude mice. After 24 h, the mice were anesthetized with isoflurane and euthanized by cervical dislocation. Hearts, livers, spleens, lungs, and kidneys were collected and stained with H&E staining reagents. The results are shown in **Figure 9**. No significant difference was observed in the pathology images of important organs between the Control group and the Rg1 group. The data above also indicated that ginsenoside Rg1 exhibited high biosafety *in vivo*, which is desirable for application in future anti-cancer clinical adjuvant therapy.

## 4 Discussion

In the clinic, cancer patients often experience strong, stressful emotions upon being diagnosed with cancer. In 2020, our research group published an article that revealed the inhibitory effects of mental stress on the anti-cancer function of human granulocytes (28). Data showed that stress hormones (hydrocortisone, adrenaline, and NA) are released into the peripheral blood under mentally stressful conditions. These stress hormones could further inhibit the anti-cancer function of human granulocytes and promote the occurrence and development of cancer.

Granulocytes include neutrophils, eosinophils and basophils (59). Neutrophil are the most abundant leukocytes in peripheral blood of healthy human (60). Eosinophils account for 0~3% of the total leukocyte count (59). The number of basophils in peripheral blood is extremely low, less than 1% of total leukocytes (59). In the study, we separated granulocytes from human blood by Percoll density gradient centrifugation. Neutrophils account for more than 95% of granulocytes obtained by this method. Therefore, the results of granulocytes in the study also mainly reflect changes of the anti-cancer function of neutrophils.

This raises the question as to how stress-induced suppression of the immune system of patients with cancer can be eliminated or relieved. To address this issue, in our previous research, NA was selected as a representative stress hormone to screen herbal extracts, which could effectively reverse the immunosuppressive effects of NA.

Ginsenoside Rg1 is the most active and abundant components of ginseng (61). Ginsenoside Rg1 has medicinal value due to their steroidal structure and exert pharmacological effects against a variety of diseases. Research shows that ginsenoside Rg1 has neuroprotective activity through inhibition of oxidative stress and neuroinflammation (62). In the cardiac-cerebral vascular disease field, ginsenoside Rg1 effectively promotes angiogenesis and attenuates myocardial fibrosis, leading to improved left ventricular function (63). Meanwhile, the great potential of ginsenoside Rg1 has been reported in clinical research against cancer. It inhibits breast cancer cell migration and invasion by suppressing MMP-9 expression and induces apoptotic cell death in triple-negative breast cancer cell lines (32, 64). Ginsenoside Rg1 also can increase the immune activity of CD4(+) T cells. However, the anti-cancer immunoprotective effect of ginsenoside Rg1 on neutrophils has not been reported. We found that ginsenoside Rg1 could effectively enhance the anti-cancer function of granulocytes inhibited by NA, which showed potential for clinical application.

In this study, CCK-8 and *ex vivo* experiments were performed to investigate the immunoprotective effects of ginsenoside Rg1 on the anti-cancer function of granulocytes inhibited by NA *in vitro* and *ex vivo*. Transcriptome sequencing analysis and qRT-PCR were used to investigate the immunoprotective mechanism of ginsenoside Rg1. Transwell cell migration experiments were performed to verify the immunoprotective effects of ginsenoside Rg1 *in vitro*. Tumor-bearing model mice were used to verify the immunoprotective effects of ginsenoside Rg1 *in vivo*. CCK-8 and H&E staining tests were performed to investigate the biosafety of ginsenoside Rg1 *in vitro* and *in vivo*. The obtained data indicated that NA exhibited significant inhibitory effects on the anti-cancer function of granulocytes, while ginsenoside Rg1 exhibited significant immunoprotective effects on the anti-cancer function of granulocytes inhibited by NA.

The immunosuppressive mechanism of NA can be described as follows: NA elevated the mRNA expression of *ARG2*, *MMP1*, *S100A4*, and *RAPSN* in granulocytes, thereby inhibiting the CKA of granulocytes and promoting cancer development. The study showed that overexpression of *ARG2* could cause immune cell dysfunction (65). *ARG2* expression was increased in prostate cancer (66, 67), breast cancer (68) and glioblastoma (69). Overexpression of *ARG2* promoted the *MMP2/9* expression, further enhancing tumor cell proliferation, migration, invasion and angiogenesis (69). *MMP1* was also overexpressed in a variety of cancers (70–72). Because of its role in extracellular matrix degradation in tumor invasion, dysregulation of *MMP1* transcription promotes tumor metastasis (73–75). Overexpression of *S100A4* promoted metastasis of non-metastatic human breast cancer cells to the lung and lymph nodes (76). The study demonstrated that macrophages, fibroblasts, and tumor cells all could release *S100A4* into the tumor microenvironment (77), and its elevated concentration promoted the formation of a pre-metastatic niche (78, 79). Several studies revealed an association between RAPSNT hypomethylation in the peripheral blood of different populations and breast and lung cancer (51, 52, 80).

The immunoprotective mechanism of ginsenoside Rg1 can be described as follows: ginsenoside Rg1 inhibits the mRNA expression of *ARG2*, *MMP1*, *S100A4*, and *RAPSN* and elevates the mRNA expression of *LAMC2, DSC2, KRT6A*, and *FOSB*, which enhance the CKA of granulocytes (which was inhibited by NA) and inhibit the development of cancer. Mature neutrophils entered the circulation from the bone marrow and migrated along a chemotactic gradient in the interstitium to perform their immune function (81). *LAMC2* could promote neutrophil chemotaxis and stimulate their motility (53, 54). Moreover, *DSC2* was an important member of the desmosomal cadherin family and served as a vital regulator in signaling processes such as migration, differentiation, and cell apoptosis (55). It was found that desmosomes are important for maintaining cell migration ability (82). *DSC2* also inhibited the metastasis of gastric cancer by inhibiting the BRD4/Snail signaling pathway and the transcriptional activity of β-catenin (83). Moreover, the loss of *DSC2* promoted the proliferation of colon cancer cells (56). The upregulation of *LAMC2* and *DSC2* expression improved the migration ability of granulocytes, which is consistent with the results of the cell migration assay in this study. The protein encoded by *KRT6A* is a member of the keratin gene family. The peptides from the C-terminal region of the protein show antimicrobial activity against bacterial pathogens. *KRT6A* protein inhibits the proliferation, migration and invasion abilities of lung adenocarcinoma cells, and high expression of *KRT6A* protein is a predictor of good prognosis in patients with lung adenocarcinoma (57). Neutrophils could directly kill tumor cells by releasing NO (84). *FosB* was a transcription factor involved in NO production through modulation of iNOS expression (58).

In summary, NA contributed to the proliferation and invasion of tumor cells, while ginsenoside Rg1 enhanced the migration capability and anti-cancer activity of granulocytes, thus inhibiting the proliferation and invasion of tumor cells.

This study highlights a new direction for the clinical application of ginsenoside Rg1 in the future. Ginsenoside Rg1 is expected to be used as an adjuvant drug treatment for patients with cancer suffering from mental stress in the future.

## Data availability statement

The transcriptome sequencing and analysis data that support the findings of this study are openly available in NCBI SRA, accession number: PRJNA929088. Other raw data that support the findings of this study are available from the corresponding author upon reasonable request.

## Ethics statement

The studies involving human participants were reviewed and approved by Tongji University Institutional Review Board (Grant No. 2019tjdx282). The patients/participants provided their written informed consent to participate in this study. The animal study was reviewed and approved by Tongji University Institutional Review Board (Grant No. TJAA07221402).

## Author contributions

BC and XH: Conceptualization, Methodology, Writing - Reviewing and Editing. YZ and XH: Investigation, Data curation, Writing - Original draft preparation. JC, JL and CZ: Software, Validation. All authors contributed to the article and approved the submitted version.
