# Supplementary material for: Ginsenoside Rg1 as a promising adjuvant agent for enhancing the anti-cancer functions of granulocytes inhibited by noradrenaline
Source: Front Immunol. 2023 Feb 1;14:1070679. doi: 10.3389/fimmu.2023.1070679 (PMC9929943; doi:10.3389/fimmu.2023.1070679)
Supplement: Supplementary file 1 [file DataSheet_1.docx]

Supplementary Material


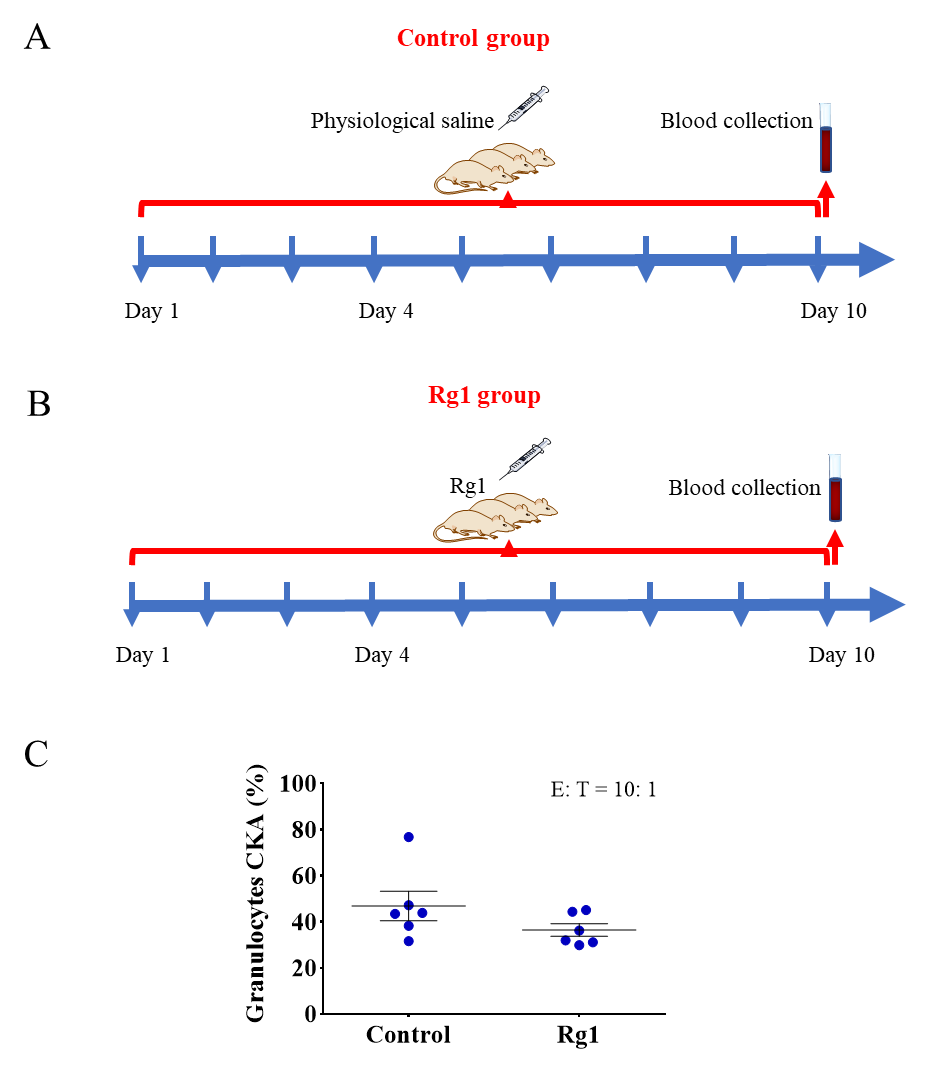


**Supplementary Figure 1.** In the *ex vivo* test, ginsenoside Rg1 exhibited no significant effect on the cancer-killing efficiency of granulocytes in healthy rats. (A - B) The detection process about the effects of ginsenoside Rg1 on the cancer-killing efficiency of granulocytes in healthy rats. (C) Effects of ginsenoside Rg1 (20 mg/kg) on the cancer-killing efficiency of granulocytes in healthy rats.


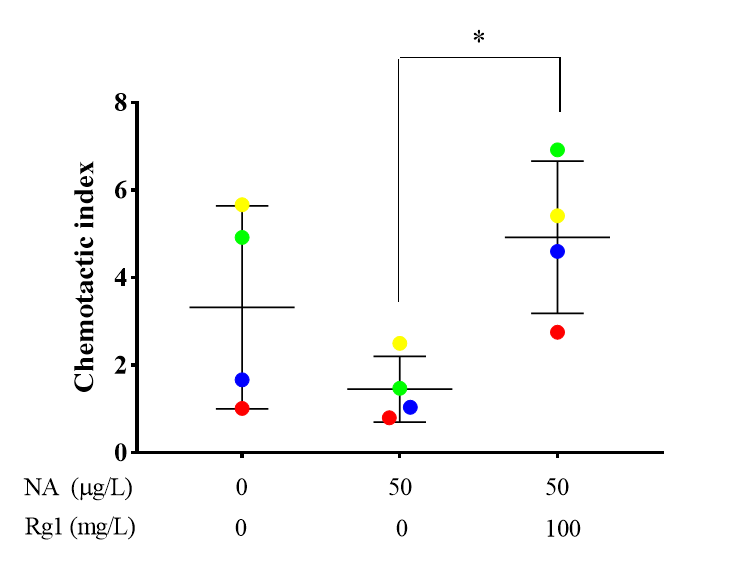


**Supplementary Figure 2.** Ginsenoside Rg1 (100 mg/L) could enhance the chemotactic function of granulocytes (which was immunosuppressed by NA) (n = 4; mean ± SD; two-tailed student’s *t*-test; *, *P* < 0.05).


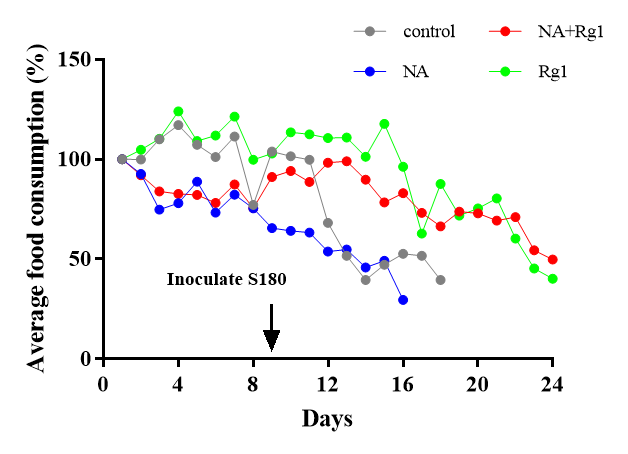


**Supplementary Figure 3.** The immunoprotective effects of ginsenoside Rg1 *in vivo*. Data of average food consumption.
